# Canonical TGFβ Signaling and Its Contribution to Endometrial Cancer Development and Progression—Underestimated Target of Anticancer Strategies

**DOI:** 10.3390/jcm10173900

**Published:** 2021-08-30

**Authors:** Piotr K. Zakrzewski

**Affiliations:** Department of Cytobiochemistry, Faculty of Biology and Environmental Protection, University of Lodz, Pomorska 141/143, 90-236 Lodz, Poland; piotr.zakrzewski@biol.uni.lodz.pl; Tel.: +48-42-635-52-99

**Keywords:** endometrial cancer, TGFβ isoforms, TGFβR1, TGFβR2, Smad proteins, TGFβ co-receptors, betaglycan, endoglin

## Abstract

Endometrial cancer is one of the leading gynecological cancers diagnosed among women in their menopausal and postmenopausal age. Despite the progress in molecular biology and medicine, no efficient and powerful diagnostic and prognostic marker is dedicated to endometrial carcinogenesis. The canonical TGFβ pathway is a pleiotropic signaling cascade orchestrating a variety of cellular and molecular processes, whose alterations are responsible for carcinogenesis that originates from different tissue types. This review covers the current knowledge concerning the canonical TGFβ pathway (Smad-dependent) induced by prototypical TGFβ isoforms and the involvement of pathway alterations in the development and progression of endometrial neoplastic lesions. Since Smad-dependent signalization governs opposed cellular processes, such as growth arrest, apoptosis, tumor cells growth and differentiation, as well as angiogenesis and metastasis, TGFβ cascade may act both as a tumor suppressor or tumor promoter. However, the final effect of TGFβ signaling on endometrial cancer cells depends on the cancer disease stage. The multifunctional role of the TGFβ pathway indicates the possible utilization of alterations in the TGFβ cascade as a potential target of novel anticancer strategies.

## 1. Introduction

Endometrial cancer is one of the most commonly diagnosed gynecological tumors. According to GLOBOCAN 2020 data, endometrial cancer is diagnosed in around 417,367 women worldwide, and is the cause of almost 97,370 deaths each year, with the world morbidity around 8.7/100,000 of the female population. Overall lifestyle risk factors of endometrial cancer determine its higher incidence rate among women in developed countries in contrast to less-developed ones [1]. What is more, in the future, the incidence of endometrial cancer is expected to increase due to the gradual aging of the population, as this tumor type occurs predominantly in women in their menopausal and postmenopausal age. Endometrial cancer is commonly classified into type I endometrioid and type II non-endometrioid, and this division is based on the clinicopathological features and different pathogenesis. Type I is the most often diagnosed type of endometrial cancer (75–90%) which develops from glandular cells in the endometrium lining. Endometrioid tumors are estrogen-dependent and tend to be low grade with a favorable prognosis. Type I is predominantly represented by endometrial adenocarcinomas, whereas non-endometrioid cancers typically include papillary serous or clear cell carcinomas, in general, histological subtypes characterized by more aggressive phenotypes with poor outcome [2,3]. FIGO I and II, early stages of endometrial cancer, are mostly diagnosed in 75% of patients. In these stages, 5-year overall survival ranges from 74% to 91%, whereas more advanced stages, i.e., FIGO III and FIGO IV are characterized by 57–66% and 20–26% 5-year overall survival rates, respectively [4].

Along with the progress in molecular biology, the above-mentioned classification of endometrial cancer, proposed by Bokhman in 1983, is extended now by molecular findings based on a large-scale, comprehensive genetic analysis of endometrial cancer according to The Cancer Genome Atlas. Molecular classification of endometrial cancer includes four subgroups, i.e., DNA polymerase epsilon ultramutated (POLE), microsatellite instability-high (MSI-H), copy-number low, and copy-number high subgroup. Each subgroup is characterized by distinct clinical, pathological, and molecular aberrations. The POLE subgroup displays polymerase epsilon mutations in the exonuclease domain, which results in a remarkably high mutation rate (232 × 10^−6^ mutations per Mb). The MSI subgroup is related to deficiencies in a DNA mismatch repair system leading to common mutations of ARID5B, PTEN, PIK3CA, and PIK3R1 genes. The copy-number low subgroup is described also as microsatellite stable and corresponds to more than half of low-grade endometrioid tumors, whereas the copy-number high subgroup reflects serous histopathology [5,6,7]. In contrast to sporadic endometrial cancer, up to 5% of tumors are described as familial ones, due to the loss-of-function or expression alterations of DNA mismatch repair genes, i.e., MLH1, MSH2, MSH6, or PMS2. The most frequent familial form of endometrial cancer is associated with Lynch syndrome (also called hereditary nonpolyposis colorectal cancer, HNPCC), which increases the risk of developing this tumor type, depending on the study, in the range of 25–60% [8,9].

To date, there are identified and well-studied lifestyle and socio-demographic risk factors underlying the development of endometrial cancer. The important risk factors for uterine carcinogenesis are obesity, the onset of menarche, reproductive history, ethnicity, and patient’s age [10,11,12,13,14,15,16]. Overweight, young age at menarche, or nulliparity are associated with prolonged exposure to estrogens. Estrogens are the major female sex hormones of high proliferative potential for uterus lining. In obese women, unopposed estrogen stimulation is a consequence of reduced progesterone synthesis and higher levels of circulating estrogens. Pregnancy is a natural period in women’s life when estrogen exposure is shifted toward progesterone. Furthermore, as it has been observed, an increased number of births has a protective effect minimizing endometrial cancer risk, in contrast to nulliparous women, who are characterized by a high occurrence of uterine neoplastic lesions [16,17,18].

The deregulation of signal transduction underlies many human diseases, in particular cancer. Impaired signaling is responsible for unsettled genes expression, which results in neoplastic transformation of affected cells. Many disturbances were identified in divergent signaling pathways which seem to play a crucial role in the origin, development, and metastasis of tumors. One of them is the cascade induced by a diverse set of proteins known as transforming growth factors β type superfamily (TGFβ), whose name comes from TGFβ itself and their isoforms serve as prototype molecules. Transforming growth factor β (TGFβ) is a large superfamily of cytokines that control a plethora of molecular and cellular processes. Up until now, the superfamily comprised more than 40 different ligands that included TGFβ itself as well as bone morphogenetic proteins (BMPs), activins, inhibins, nodal growth differentiation factor (Nodal), growth and differentiation factors (GDFs), left-right determination factor (Lefty), and anti-Müllerian hormone/Müllerian inhibiting substance (AMH/MIS). TGFβ ligands are key regulators during embryonic development, tissue formation and regeneration, and their alteration results in body malformation, cancer development and progression, fibrosis, and autoimmunological diseases [19,20,21,22]. This review is focused on TGFβ ligands as prototypical TGFβ superfamily members and their role in the development and progression of endometrial cancer.

The inclusion criteria of literature selected for this review included the search in PubMed of the National Library of Medicine using the following keywords: “TGFβ1-3” or “TGFβR1” or “TGFβR2” or “Smad1-7” or “betaglycan/TGFβR3” or “endoglin/CD105” and “human endometrium” or “endometrial cancer”. Neither of the selected articles was published in languages other than English nor were retracted from publication. The relevance of the articles was evaluated by analyzing the title and abstract. The potentially relevant articles were full-text evaluated and included in this review.

## 2. TGFβ Ligands—Synthesis, Secretion, and Activation

The TGFβ superfamily consists of 33 ligands that possess cytokine activity. A large number of TGFβ ligands determines the plethora of processes engaged in regulation and mediation of cellular homeostasis, including embryonic development, cell differentiation and proliferation, immune response, angiogenesis, motility, and apoptosis, both in a spatiotemporal manner, as well as dependent on cell and tissue type [22,23,24,25]. All TGFβ ligands share structural homology ranging between 60% and 80%, and evolutionarily conserved motif containing from 6 to 12 cysteine residues organized in cysteine knot (CK), which is responsible for TGFβ homo- and heterodimerization. The structural similarities within the CK sequence allowed to distinguish two subfamilies among TGFβ superfamily ligands [26,27]. The first one contains prototypical three TGFβ isoforms (TGFβ1, TGFβ2, and TGFβ3), activins, nodal, lefty, and myostatins, whereas in the second one, BMPs, GDFs, and AMH/MIS can be clustered [27,28,29,30].

**Figure 1:**
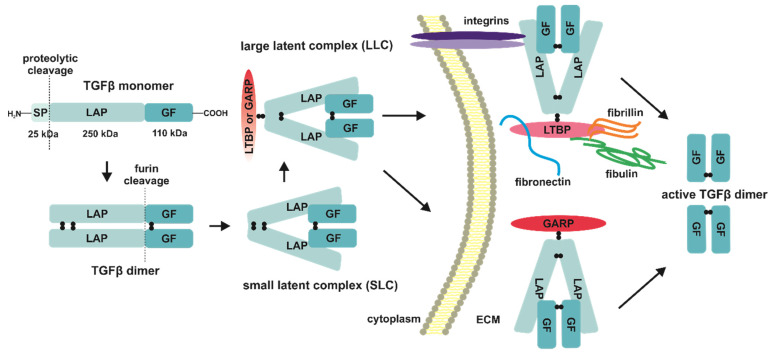
TGFβ isoforms processing, secretion, deposition, and activation. A series of molecular events is presented from left to right and guided by black arrows. The TGFβ ligands are synthesized as precursors—pre-proproteins. After biosynthesis and proteolytic cleavage of SP, two TGFβ monomers aggregate noncovalently with the following disulfide bonds formation between LAP prodomains and GF domains of each monomer. The next step in the TGFβ ligands biosynthesis is the removal of LAP prodomains by furin proteases. The LAP peptides stay noncovalently bound to pro-TGFβ dimer, and that bimolecular aggregate is called SLC. The secretion of native TGFβ ligands is preceded by the interaction of SLC with LTBP or GARP, forming the LLC. LTPB or GARP proteins being disulfide-bound to LAP determine temporary inactivation of LLC by increasing its half-life and sequestration of TGFβ dimers in extracellular space after secretion, through the interactions with fibrillin, fibronectin, or fibulin. The C-terminus of LAP also allows its binding with integrins. SP: signal peptide; LAP: latency-associated peptide prodomain; GF: growth factor; LTPB: latent TGFβ-binding protein; GARP: glycoprotein-A repetitions predominant protein; ECM: extracellular matrix.

The TGFβ ligands are synthetized as precursors—pre-proproteins consisting of an app. 29-residues secretion signal peptide (SP), an app. 250-residue latency-associated peptide prodomain (LAP), and an app. 110-residue C-terminal growth factor (GF) domain [31,32,33,34,35,36] (Figure 1). After biosynthesis and proteolytic cleavage of SP, two TGFβ monomers aggregate noncovalently in the endoplasmic reticulum (ER) with the following disulfide bonds formation between LAP prodomains and GF domains of each monomer, forming together disulfide-linked inactive dimers. The next step in the TGFβ ligands biosynthesis is the removal of LAP prodomains by furin proteases. The LAP peptides stay noncovalently bound to pro-TGFβ dimer, and that bimolecular aggregate is called a small latent complex (SLC) (Figure 1). The secretion of native TGFβ ligands is preceded by the interaction of SLC with latent TGFβ-binding protein (LTBP); what results in the formation of a so-called large latent TGFβ complex (LLC). LTPB protein being disulfide-bound to LAP determines temporary inactivation of LLC by increasing its half-life and sequestration of TGFβ dimers in extracellular space after secretion, through the interactions with matrix components, i.e., fibrillin 1, fibronectin, or fibulin. Moreover, the RGD (Arg-Gly-Asp) motif present in the C-terminus of LAP allows its binding with integrins [37,38,39,40] (Figure 1). In addition to LTBP, latent TGFβ ligands can also interact with another “milieu” molecule known as glycoprotein-A repetitions predominant protein (GARP) (Figure 1). The GARP protein, also referred to as LRRC32 (leucine-rich repeat-containing 32), is a cell surface receptor identified on regulatory T-lymphocytes (Treg), platelets, hepatic stellate cells, and certain cancer cells. Its function, similar to LTBP and described at the end of the first decade of the 21st century, is binding and accumulation of latent TGFβ before the activation and release of the native cytokine. The latent states prevent uncontrolled activation of the cognate TGFβ receptors [41,42,43,44].

For full bioavailability the latent TGFβ needs several mechanisms, among which can be distinguished the proteolysis of LLC, low pH in the extracellular matrix, reactive oxygen species (ROS), as well as thrombospondin-1, retinoic acid or basic fibroblast growth factor (bFGF) dependent activation [45,46]. The main proteases responsible for the degradation of LLC are plasmin, matrix metalloproteinases 1 and 9 (MMP-1, MMP-9), or bone morphogenetic protein 1 (BMP1), which despite the same abbreviation as BMPs belonging to the TGFβ superfamily, is not related to them. The mature ligands processing may also demand the mechanical traction of specific integrin αVβ6 responsible for their interaction with extracellular matrix (ECM), whereas proteolytic cleavage can be mediated by furin-like proteases [47,48,49,50,51,52,53] (Figure 1).

## 3. TGFβ Signaling Cascade

### 3.1. TGFβ Isoforms and Their Dedicated TGFβ Receptors

The canonical TGFβ signaling in contrast to other non-canonical TGFβ induced pathways occurs upon ligand binding by specific transmembrane receptors, which possess serine/threonine kinase activity and can be divided according to their structural features into two types, i.e., type I (TGFβRI) and type II (TGFβRII). Both types of TGFβ receptors are transmembrane kinases, with an app. 100-residues ectodomain, which is highly glycosylated and disulfide-rich, a transmembrane region, a short juxtamembrane domain, and cytoplasmic kinase domain with 11 subdomains organized in N-lobe and C-lobe [25,54,55]. The presence of a 30-residues regulatory motif rich in glycine and serine (GS motif) and located upstream of the kinase domain within a juxtamembrane domain, sets TGFβRI apart from TGFβRII [56,57,58,59]. The type I TGFβ receptors are encoded by seven genes and their protein products are commonly known as activin-like kinases (ALK1-7) with the exception of ALK5, also termed as TGFβR1; whereas the human genome encodes five type II receptors abbreviated as ActRIIa, ActRIIB, BMPRII, AMHRII, and TGFβRII, respectively. The type I and type II receptors display different affinity to TGFβ ligands, hence the prototypical TGFβ isoforms (TGFβ1-3) operate only through ALK1, ALK2, ALK5 (TGFβR1), and TGFβR2. In the absence of native TGFβ dimers, the type I and type II receptors can exist as monomers, homodimers, and heterodimers at the plasma membrane, thereby preventing ligand-independent signal activation [60,61,62]. However, the combinations of ectodomains of both TGFβ receptor types, which form the heterodimers mentioned above, enable TGFβ factors selective and specific binding, and regulation of opposite cellular processes in tissues in a context-dependent manner [63,64].

### 3.2. Signal Propagation in Canonical TGFβ Pathway

**Figure 2:**
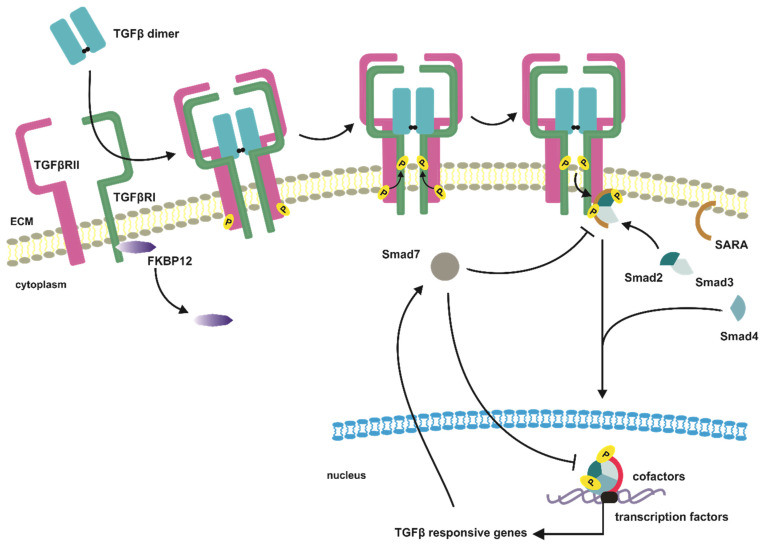
Canonical TGFβ pathway. The first step in TGFβ canonical pathway is an interaction of the dimeric TGFβ ligand with a tetrameric receptor complex consisting of two pairs of TGFβRIs and TGFβRIIs. The activated dimer of TGFβRIIs due to its constitutive kinase activity phosphorylates TGFβRIs. The ligand-induced activation of TGFβRIIs with following phosphorylation of TGFβRIs results in release from TGFβRI an inhibitory FKBP12 protein, what promotes the activation of TGFβRI kinase domains. The signal propagation occurs when activated TGFβRs tetramer phosphorylates cytoplasmic Smad2/3 proteins complex. The SARA protein stabilizes the interaction of Smad2/3 complex with TGFβRIs. An activated Smad2/3 complex translocates to the nucleus, and together with Smad4 and other transcription factors and/or cofactors, activate an expression of TGFβ responsive genes. Smad7 antagonizes TGFβ induced signaling by an association with the activated TGFβRI receptor, thus blocking an interaction, activation, and phosphorylation of Smad2 effectors. Smad7 can also disrupt the Smad-DNA complex formation in the nucleus, thereby altering cell responsiveness to TGFβ at the transcriptomic level. FKBP12: immunophilin 12-kD FK506 binding protein; SARA: Smad anchor for receptor activation.

The first step in TGFβ canonical pathway is an interaction of the dimeric ligand with a tetrameric receptor complex consisting of two pairs of TGFβRIs and TGFβRIIs. This interaction provides conformational changes of ectodomains of TGFβ receptors and brings intracellular domains to close proximity; thus, the activated dimer of TGFβRIIs due to its constitutive kinase activity phosphorylates GS motifs of the type I receptor dimers [58,65,66,67] (Figure 2). The ligand-induced activation of TGFβRII with following phosphorylation of TGFβRI results in conformational changes of cellular domains of both receptors and release from TGFβRI GS motifs inhibitory FKBP12 protein (immunophilin 12-kD FK506 binding protein), which promotes the activation of TGFβRI kinase domains [68,69,70]. The activation of the TGFβRI receptors is a complex process pronounced by the fact that the TGFβRIs cannot bind TGFβ dimers separately, what prevents from incidental initiation of TGFβ signaling in the absence of ligands (Figure 2).

Successfully transduced through the plasma membrane signal activates in turn cascade of transphosphorylation reactions of TGFβ effector proteins [71]. The signal propagation occurs when activated TGFβRs tetramer phosphorylates cytoplasmic Smads proteins (similar to mother against, mother against decapentaplegic homologs). In human cells three subclasses of Smad proteins have been identified according to their function in signal mediation, i.e., R-Smads (receptor-activated Smads—Smad1,2,3,5,8), Co-Smad (common-mediator Smad—Smad4), and I-Smads (inhibitory Smads—Smad6,7) [72,73]. The first Smads engaged in intracellular propagation of TGFβ induced signal are Smad2 and Smad3, which in dimeric form of Smad2/3 are recruited by TGFβ receptors tetramer and are transphosphorylated at their C-terminal serine residues by TGFβRI receptors [74,75,76]. The activated Smad2/3 heterodimer dissociates immediately from the membrane receptor complex and interacts with Smad4. The Smad2/3-Smad4 complex, after translocation to the nucleus, in cooperation with other transcription factors, coactivators, and corepressors, modulates the expression of TGFβ responsive genes [25,54,77] (Figure 2).

### 3.3. Smad Proteins—Structure and Function

The R-Smads assemblage to the TGFβRs complex and their activation can be mediated by chaperon proteins, such as SARA (Smad anchor for receptor activation) or ERBIN (Erbb2-interacting protein) [55,63,78]. The SARA protein is a membrane-anchored protein belonging to the FYVE domain protein family (FYVE abbreviation stands for name homology with Fab1, YOTB, Vac1, and EEA1 proteins). It facilitates and stabilizes the Smad2/3 complex with type I TGFβ receptors, thus promoting Smad2/3 complex activation [79,80] (Figure 2). The similar function in R-Smad/TGFBRI interaction plays Hgs/Hrs (hepatic growth factor-regulated tyrosine kinase substrate), which besides facilitation of the above-mentioned complex formation, cooperates with SARA to promote TGFβ-induced signal propagation [81]. In the case of ERBIN protein, its function in the context of the TGFβ pathway is to block Smad2/3 hetero-trimerization with Smad4, thus blocking TGFβ target genes [82].

**Figure 3:**
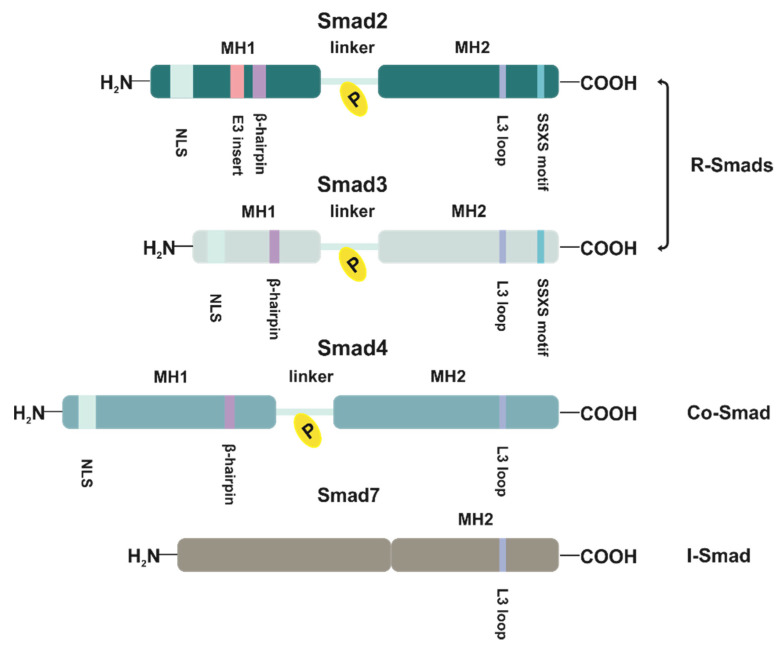
Smad proteins engaged in the canonical TGFβ pathway. Schematic representation structure of receptor-activated Smads (R-Smads), common-mediator Smad (Co-Smad), and inhibitory-Smad (I-Smad) necessary for TGFβ isoforms-induced canonical signaling. MH1 and MH 2: Mad homology 1 and 2 domains; NLS: nuclear localization signal; SSXS: Ser-Ser-X-Ser motif.

The expression of Smads-dependent genes occurs through highly conserved MH1 and MH2 (Mad homology 1 and 2) domains separated by a serine-proline rich linker of high variability [73] (Figure 3). The MH1 and MH2 domains are critical for both Smads’ interaction with TGFBRI and their function as transcriptional factors. The MH1 domain located within Smads N-terminus contains NLS sequence (nuclear localization signal) and β-hairpin structure, which is responsible for DNA sequence recognition, whereas the MH2 region promotes protein–protein interface [83,84]. The region engaged in Smads heterotrimerization and Smads/TGFBRI complex formation is located in the C-terminal fragment of the MH2 domain and is organized as an L3 loop [85,86] (Figure 3). In the case of Smad4, the L3 loop determines its assemblage to Smad2/3 [87]. Moreover, at every C-terminus of Smad2 and Smad3 MH2 domains, there is located SSXS (Ser-Ser-X-Ser) motif necessary for activating phosphorylation by activated TGFBRI [74,76] (Figure 3). In turn, the linker region contains several phosphorylation sites for kinases related to both TGFβ signaling and other pathways [88,89]. In phosphorylated states, these sites act as docking sites for other proteins, in consequence regulating Smads activity, and affecting not only protein–protein interactions but also MH1 and MH2 activity and function. The character of the phosphorylation-related effect depends on the phosphorylation pattern of the linker region [90,91,92,93,94]. Given together, the phosphorylation of Smads beyond the SSXS motif is an additional significant and fine-tuned interface for TGFβ crosstalk with other cellular pathways.

The DNA recognition by the heterotrimeric complex of R-Smads and Smad4 occurs effectively, but with low affinity by its direct interaction with the major groove of specific DNA sequences described as SBEs (Smad binding elements) [77,95]. In the case of Smad3 and Smad4, but not the most prevalent Smad2, which possesses a unique 30 amino acids sequence (E3 insert) proximal to β-hairpin and diminishing DNA binding capacity by Smad2, DNA recognition is strictly limited to the palindromic sequence 5′-GTCTAGAC-3′ [83,95,96] (Figure 3). Furthermore, according to recent studies, the Smad3 and Smad4 are also able to bind 5-bp GC-rich regulatory elements of 5′-GGC(GC)|(CG)-3′ consensus sequence [97]. However, in the light of new experimental data, the lack of Smad2 DNA binding activity seems to be not so evident and can depend on the conformational state of E3 insert. In the open conformational state of E3 insert, Smad2 presents DNA binding ability, whereas its close state abrogates Smad2-DNA contact [98]. As Smads complexes bind DNA with relatively low affinity (Kd = 1.18 × 10^−7^ M), it seems that modulation of TGFβ-target genes expression is shaped by the association of Smads with different transcription factors both activators and repressors, which enhance overall interaction [83]. The shape and the biological effect of these interactions are highly dependent on the transcription factor type, tissue specificity, or physiological state of the cell [99]. It can occur according to the different scenarios, either high-affinity transcription factors recruit activated Smads, or they bind cooperatively with Smads to a consensus sequence. Nonetheless, these mutual interactions determine the increased affinity of each component to DNA and lead to activation or repression of Smad-responsive genes [77,100].

The specific role in TGFβ canonical pathway is played by Smad7 protein, which belongs to the subclass of I-Smad (Figure 3). The main function of Smad7 is to antagonize TGFβ induced signaling by association with activated TGFβRI receptor, what results in blocking of interaction, activation, and phosphorylation of Smad2 effectors [101] (Figure 2). Moreover, in the nucleus, Smad7 can also disrupt the Smad-DNA complex formation, thereby altering cell responsiveness to TGFβ at the transcriptomic level [102,103]. The Smad7 is also an essential element of the TGFβ negative feedback loop, as SMAD7 is a target gene of TGFβ signaling [104,105,106] (Figure 2). The TGFβ ligands promote the cytoplasmatic localization of Smad7, which in the absence of ligands is predominantly present in the nucleus [107].

The negative regulation via Smad7 protein can also occur via mobilization of E3 ubiquitin ligases, i.e., SMAD ubiquitination regulatory factor 1 and 2 (Smurf1 and Smurf2), WW domain-containing protein 1 (WWP1), and neural precursor cell expressed developmentally downregulated 4-2 (NEDD4-2), which in turn promote ubiquitin-dependent proteasomal and/or lysosomal degradation of TGFβRI [108,109,110,111,112]. Furthermore, NEDD4-2 ubiquitin-mediated degradation may also include Smad2, whereas, in the case of Smurf1, it affects Smad4 when in ternary complex with Smurfs and Smad2 or Smad6/7, acting as adaptors [110,113]. The Smad7 negative regulation of TGFβ-induced signaling is kept in reciprocal balance, as TGFβ facilitates the expression of transforming growth factor-β-stimulated clone 22 (TSC-22), which effectively competes with Smad7 for TGFBRI binding, in effect preventing TGFβRI from degradation [114].

### 3.4. TGFβ Receptors Trafficking, Internalization, and Recycling

To fully understand the TGFβ receptors’ functioning and their engagement in signal propagation, it is necessary to focus on another important issue connected with TGFβRs activity and cellular metabolism, i.e., trafficking, internalization, and recycling [115]. In contrast to the many other signaling receptors, TGFβRs are predominantly expressed intracellularly and are translocated to the cell surface upon TGFβ stimuli to provide an interface for TGFβ ligands stimulation. Both TGFβ receptors are synthetized in ER with the following posttranslational modifications in the Golgi apparatus prior to being transported to the cell membrane [116,117]. The TGFβRs processing in the Golgi apparatus includes N-linked glycosylation and is necessary for successful plasma membrane transportation [118,119]. The matured TGFβ type I and type II receptors are in turn translocated to the cell surface from the trans-Golgi network (TGN), however, its route depends on the receptor type. The TGFβRII-containing post-Golgi vesicles are effectively delivered to the cell surface via its interaction with microtubules, traveling along with them to the plasma membrane [120,121]. On the other hand, the translocation of TGFBRI from intracellular stores is mediated by Akt-dependent phosphorylation of Akt substrate of 160 kDa (AS160) [122]. The AS160, which contains the GTPase-activating protein (GAP) domain, is engaged in activation of small G-proteins, i.e., Rab2, Rab8, Rab10, and Rab14, and phosphorylation by Akt inhibits its GAP activity [123]. The Rabs enhance the transportation of proteins, including TGFβRI, between intracellular compartments and cell surface, due to their participation in vesicle movement and fusion [122]. The Rab2 is present in pre-Golgi intermediates and is involved in proteins migration from the ER to the Golgi complex [124]. The Rab8 is responsible for vesicular trafficking from the TGN to the basolateral plasma membrane [125]. The ER structure and dynamics are controlled by Rab10 [126], while the biosynthetic/recycling pathway between the Golgi and endosomal compartments is under the control of Rab14 [127].

While the native TGFβRs are delivered to the plasma membrane, they organize in a well-defined distribution pattern dependent on the cell type. In polarized epithelial cells, TGFβRs localize to the basolateral plasma membrane, where they are involved in TGFβ-induced signaling, leaving the apical side insensitive to TGFβ stimulation [128,129,130,131]. On the contrary, non-epithelial or non-polarized epithelial cells are characterized by a dispersed manner of the TGFβRs expression, though the higher concentration of TGFβRs is observed in membrane ruffles, sites of cell–cell contact, and at the leading edge of migrating cells [132,133]. It has been shown that elements crucial for basolateral expression of TGFβRs are amino acids motifs located between residues 158–163 (VxxEED) of TGFβRI and 529–538 (LTAxxVAxxF) of TGFβRII [129,131]. It is worth pointing out that the cell surface TGFβRs are constantly internalized, both in the presence or absence of TGFβ ligands. However, this process is not as evident as in the case of plasma membrane receptors of other signaling pathways, which internalize in the ligand-bound state to transmit signals into the cytoplasm. As observed, the TGFβRs endocytosis in response to TGFβ stimuli is not essential for TGFβ signaling, and TGFβ stimulation does not affect rates of internalization or receptors recycling to the cell surface [134,135,136]. The TGFβRs are found to have distinct localization in the plasma membrane microdomains, protruding in non-raft clathrin-coated pits and caveolin-1 positive cholesterol-rich lipid rafts [137].

The clathrin-coated pits are microdomains in the plasma membrane covered at the cytoplasmatic side with clathrin triskelions, and they are engaged in cargo enrichment and endosomal vesicles formation [138,139]. The elements responsible for TGFβRI and TGFβRII clathrin-dependent endocytosis are di-leucine motifs in the cytoplasmic regions proximal to the transmembrane domains of both receptors, respectively Leu180-Ile181 for TGFβRI and Ile218-Ile219-Leu220 for TGFβRII [140,141]. The clathrin-mediated internalization into an early endosome antigen (EEA)-1-positive endosome promotes canonical signaling by increasing SMAD2 nuclear translocation and thereby activating downstream signaling [137].

The caveolar internalization occurs in the presence of flask-shaped membrane invaginations organized in lipid rafts of cholesterol and sphingolipids microdomains and enriched by caveolin-1 protein [142]. The caveolin-1 binds the TGFβRI via the scaffolding domain of caveolin-1, while the determinant of TGFβRII partitioning into lipid rafts and receptors interaction with caveolin-1 is assigned to the extracellular domain of the TGFβRII [143,144]. In contrast to clathrin-mediated internalization, the caveolin-dependent endocytic pathway functions as TGFβ signaling “turn off”, thus suppressing TGFβ-induced Smad2 phosphorylation and following downstream events [143]. The above-mentioned signal inhibition can be a result of diminished activity of TGFβRI due to the caveolin-1 binding to its kinase domain [143]. There is also a report concerning the role of caveolar endocytosis in promoting Smad7/Smurf1/2-dependent TGFβ receptors degradation [137]. Given together, both non-lipid and lipid raft trafficking pathways can compete for TGFβRs sequestration, thus keeping the balance between signal transduction and receptors turnover.

The partitioning of TGFβRs in the cell membrane determines the fate of TGFβ-induced signal or TGFβRs themselves [135]. Furthermore, TGFβRs distribution undergoes dynamic fluctuations, since it is constantly modulated by the chemical composition of the plasma membrane, interaction with various auxiliary proteins, extracellular stimuli, or posttranslational modification of TGFβRs themselves [135,139,145,146]. The IL-6 stimulation promotes recruitment of the TGFβRs to the non-lipid raft fraction following an increase of TGFβ signaling in human renal proximal tubular epithelial cells [147]. Unlike IL-6, the exogenous hyaluronan through its receptor CD44 facilitates TGFβRs trafficking into caveolin-1 lipid raft-associated pools in MAP kinase-dependent manner, in effect augmenting TGFβRs turnover [148]. The heparan sulfate is another polysaccharide, which can shift the TGFβRs to the lipid raft fraction [149]. The selective endocytosis of TGFβRII receptors is observed in the case of a disintegrin and metalloproteinase 12 (ADAM12), which not only favors TGFβRII localization into clathrin-coated pits in protease-independent mechanism but also antagonizes Smad7 suppressing impact on TGFβRs via accumulation of TGFβRII in early endosomal vesicles [150]. The endocytic protein disabled-2 (Dab2) enhances clathrin-mediated lateral diffusion of TGFβRI in the plasma membrane, as well as TGFβRs complex internalization [151,152]. The assemblage of TGFβRII to clathrin-positive pits instead of caveolae is also promoted by neddylation (conjugation of neural precursor cell-expressed, developmentally downregulated 8 (NEDD8) moieties) of TGFβRII at positions of Lys556 and Lys567. This ubiquitin-like modification is mediated by Casitas B-lineage lymphoma (c-Cbl), a known proto-oncogene encoding a ubiquitin E3 ligase [153].

## 4. TGFβ Co-Receptors

### 4.1. TGFβ Signaling Is Modulated by TGFβ Co-Receptors

In the mid-1980s, the third type of TGFβ receptor was identified and termed in the literature as the TGFβ receptor type III (TGFβRIII). Other commonly used terms for this type of receptor are accessory/auxiliary receptors or co-receptors. The name of the TGFβ receptor type III originates from the common function shared by its members, not from their structural similarities. These receptors play a vital role in presenting TGFβ ligands to the signaling receptors, enhancing receptor–ligand interactions, and promoting the cooperation between canonical TGFβ receptors. Structurally, TGFβ co-receptors are transmembrane proteins or proteins anchored in a membrane by glycophosphoinositol (GPI). Unlike TGFβRI and TGFβRII, they are deprived of any known intrinsic motifs possessing enzymatic activity. Some co-receptors mediate ligand binding by increasing its affinity, whereas the others provide an interface for structural changes to allow ligand–receptor interaction. Furthermore, co-receptors ectodomain shedding delivers extracellular molecules responsible for TGFβ ligands sequestration in ECM, thus modulating or antagonizing TGFβ responsiveness at the signal initiation step [31,54,64].

To date, a few molecules have been identified and described as acting as TGFβ co-receptors, including betaglycan (TGFβR3), endoglin (CD105), CD109, and repulsive guidance molecules a-c (RGMa, RGMb, RGMc), in general, well-known and extensively studied TGFβ receptors type III [18,154,155,156,157]. Recently however, the term “TGFβ co-receptors” has been modified, as there have been described novel proteins or cellular components modulating TGFβ response, as well as providing possible ways for integration of TGFβ canonical pathway with the other signaling cascades. According to that, the TGFβ co-receptors include, for example, neuropilins, which link TGFβ route with vascular endothelial growth factor (VEGF) signaling; the integrins, which are involved in TGFβ ligands activation; the muscle-specific kinase (MuSK) belonging to the receptor tyrosine kinases and operates as BMPs co-receptor; the BMP and activin membrane-bound inhibitor (BAMBI); or the so-called SCUBE proteins (signal peptide-complement protein C1r/C1s-Uegf-BMP1-EGF domain-containing proteins) [158].

### 4.2. Betaglycan and Endoglin Structure and Function

**Figure 4:**
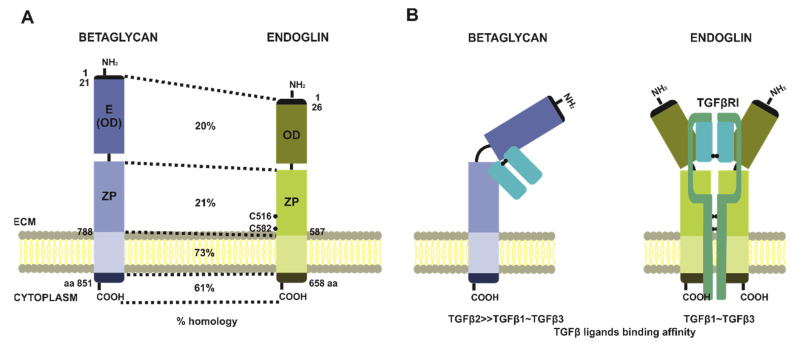
Comparison of TGFβ co-receptors i.e., betaglycan and endoglin domain structure homology (**A**) and conformational changes upon TGFβ dimers binding (**B**); E: endoglin related domain; OD: orphan domain; ZP: zona pellucida domain; ECM: extracellular matrix.

Among the above-mentioned TGFβ co-receptors, only betaglycan, endoglin and CD109 participate in a modulation of the signal induced by three TGFβ isoforms. Although, betaglycan and endoglin are not only structurally related but also of the highest importance for cancer development and progression [18,154,155,156,157,158] (Figure 4A). Betaglycan was the first identified molecule with an assigned function as a TGFβ co-receptor [159]. It is a transmembrane proteoglycan sharing structural homology with another co-receptor—endoglin [160,161]. Both co-receptors are homologs within the whole sequence, with particular regions of variability. The general domain structure includes an N-terminal signal peptide, a large ectodomain, a single-spanning transmembrane helix, and an app. 40-residues short cytoplasmic tail. The highest homology is observed in the case of cytoplasmic and transmembrane fragments, respectively at the level of 61% and 73%, whereas two distinct regions of ectodomains show only 20–21% of homology [162,163,164]. Nevertheless, the overall structure of extracellular regions of betaglycan and endoglin is similar, and within their sequences, we can distinguish unique membrane-distal N-terminal halves (OD stands for an orphan domain), and membrane-proximal one, which is characteristic for zona pellucida family of proteins (ZP domain-containing proteins) [163]. Furthermore, these two subdomains are important elements determining betaglycan’s and endoglin’s ability to interact with TGFβ ligands. The amino-terminal end of betaglycan is sometimes referred to in the literature as endoglin-related [165] (Figure 4A). What differs both co-receptors are their organization in the plasma membrane, where betaglycan is present and acts as a monomer, while endoglin undergoes homodimerization via disulfide bond created between cysteine residues and located in ZP domains. This fact reflects in a stoichiometry of TGFβ dimers binding. Betaglycan interacts with ligands asymmetrically with a ratio of 1:1, where ZP and OD domains are embracing TGFβ dimer [166] (Figure 4B).

Endoglin binding of TGFβ homodimers can be predicted based on its interaction with BMP9 or BMP10. According to these findings, endoglin might bind TGFβ homodimers with 2:1 stoichiometry, and its homodimer forms an antibody-like structure, where homodimerized fragments of ZP domains resemble the Fc region, and both OD domains of endoglin molecules serve as Fab region. In such a Y-shaped structure, TGFβ binding occurs symmetrically, with ligand binding sites located within OD domains [167] (Figure 4B). Despite the structural similarities, betaglycan and endoglin bind TGFβ dimers with different specificity and affinity, and also show preferential tissue expression [158,168]. In contrast to endoglin, whose distribution is limited to endothelial cells, betaglycan is much more ubiquitous, and its altered expression is observed in different diseases, particularly carcinomas. On the other hand, endoglin is considered a remarkable angiogenic factor whose presence contributes to the development of tumor vasculature [168,169,170].

As it has been shown, betaglycan can form stable binary complexes with all three TGFβ isoforms, i.e., TGFβ1, TGFβ2, and TGFβ3, though with the preferential complex formation with TGFβ2, which has 200–500-fold weaker affinity for its cognate TGFβRII, as compared to TGFβ1 and TGFβ3 [161,171]. Interestingly, this suggests that betaglycan acts as an essential player, responsible for recruitment, presentation, and signal propagation, increasing TGFβ2-induced responsiveness [165,172,173]. On the other hand, endoglin can bind only TGFβ1 and TGFβ3 isoforms, but for these interactions, the presence of TGFβRII is required [174]. Conversely, betaglycan forms complexes with TGFβ ligands regardless of the presence of TGFβRs [175].

**Figure 5:**
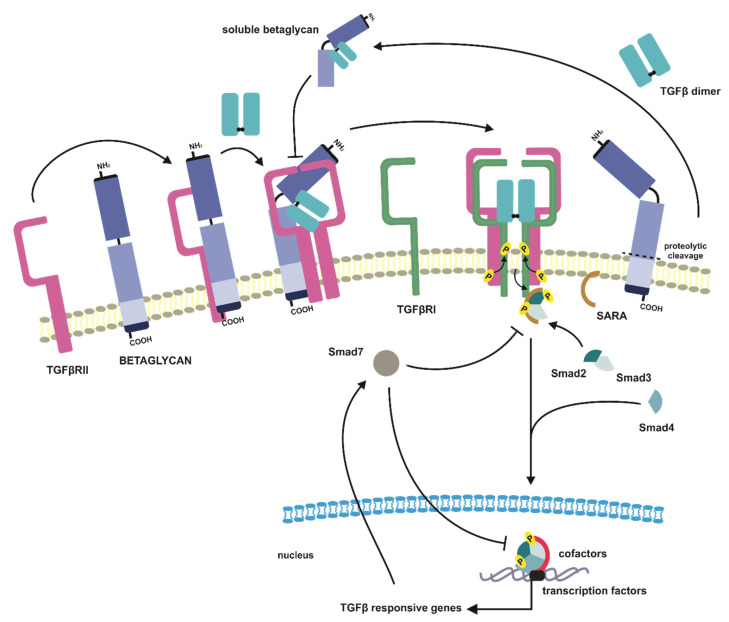
Canonical TGFβ signaling mediated by TGFβ co-receptor betaglycan and soluble betaglycan formed after proteolytic cleavage. TGFβ ligands presentation to the dedicated TGFβRs includes the formation of a binary complex between betaglycan and TGFβ dimer, which potentiates binding of TGFβRIIs. In turn, once bound, TGFβRIIs lead to recruitment of TGFβRIs, with a simultaneous displacement of betaglycan from signaling complex. Betaglycan can also modulate TGFβ signaling due to its ectodomain shedding. A soluble form of betaglycan effectively sequestrates TGFβ dimers, thus diminishing pathway activation.

**Figure 6:**
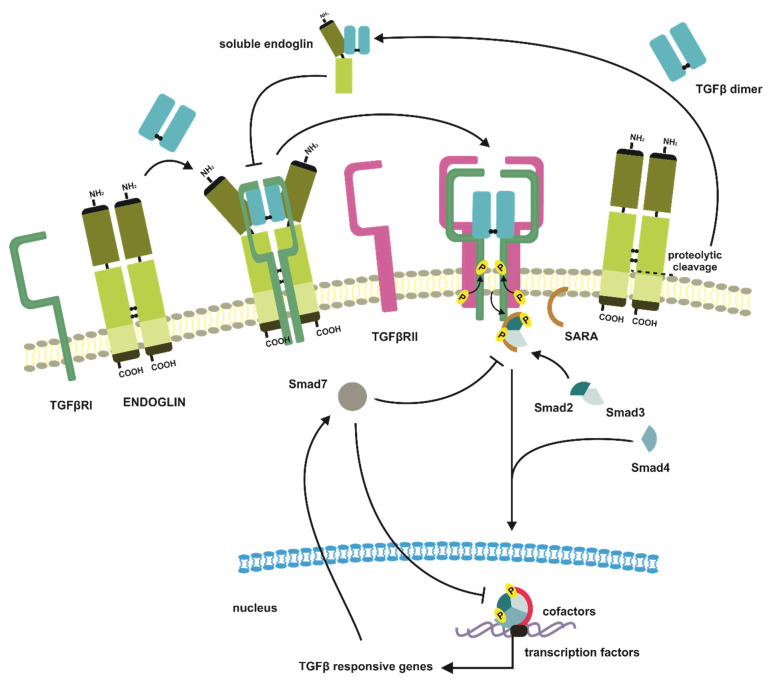
Canonical TGFβ signaling mediated by TGFβ co-receptor endoglin and soluble endoglin formed after proteolytic cleavage. In the case of endoglin, TGFβ dimers binding results in endoglin conformational changes, with sequential facilitation of TGFβRIs attachment, and endoglin dimer replacement with TGFβRIIs. Endoglin can also modulate TGFβ signaling due to its ectodomain shedding. A soluble endoglin effectively sequestrates TGFβ dimers, thus diminishing pathway activation.

The mechanistically, TGFβ ligands presentation to the dedicated TGFβRs includes the formation of a binary complex between betaglycan and TGFβ dimers, what potentiates binding of TGFβRIIs. In turn, once bound, TGFβRIIs leads to recruitment of TGFβRIs, with a simultaneous displacement of betaglycan from signaling complex [172] (Figure 5). In the case of endoglin, what was confirmed by BMP9 or BMP10 interaction studies, TGFβ dimers binding results in conformational changes, what sequentially facilitates attaching of TGFβ receptors type I (ALK1), and endoglin dimer replacement with specific for these ligands type II receptors (ActRII, ActRIIB, or BMPRII) [167,176] (Figure 6).

The TGFβ co-receptors not only act as significant molecular agents responsible for potentiation of the extracellular signal induced by TGFβ dimers but also are able to modulate TGFβ response due to their ectodomain shedding. Both betaglycan and endoglin may undergo proteolytic cleavage, releasing to the ECM their extracellular domains [177,178] (Figure 5 and Figure 6). Present in the ECM soluble forms of betaglycan and endoglin effectively sequestrate TGFβ dimers, thus diminishing pathway activation. Soluble co-receptors compete with those membrane-bound, as they display unchanged affinity to TGFβ ligands. Soluble endoglin in comparison to its membrane-bound form circulates in ECM as a monomer. The impaired generation of soluble betaglycan or endoglin appears to be engaged in the development of different pathological conditions, including cancer, preeclampsia, hypercholesterolemia, or pulmonary arterial hypertension; therefore, their serum levels could potentially serve as diagnostic or predictive factors [179,180,181,182,183,184].

## 5. Distribution of TGFβ Isoforms and Their Cognate Receptors in Normal Human Endometrium

### 5.1. The Architecture of Human Endometrium

The human endometrium is a hormone-dependent tissue, which in the literature is often described as “highly dynamic”. This feature associates with subsequent phases of proliferation, differentiation, shedding, and regeneration in a single menstrual cycle in total more than 400 times in a woman’s lifetime. From a biological point of view, the role of the endometrium is to provide an immunotolerant site for embryo implantation and its development in nurturing conditions until labor. In the structure of the human endometrium, we can distinguish two separate layers of different morphology and fate. The outermost layer of the endometrium is the functional one, which lines the uterine cavity and presents the transient physiology. Underneath the functional layer and adjacent to the myometrium, is located the permanent basal layer. The single strand of luminal epithelium, the stroma, and the superficial glands (glandular epithelium) build up the functional layer. As the functional layer covers the deeper basal one, the terminal parts of the glands are anchored in the basal layer [185]. During the menstrual cycle, the functional layer undergoes extensive structural and cytological changes in response to reproductive hormones, i.e., estrogen and progesterone secreted by ovaries. Hormonal regulation results in continuous fluctuations in endometrial thickness leading to shedding of functional layer throughout menstruation. After the menstrual shedding, the endometrium is regenerated and repaired from the basal layer in the intensive proliferative phase orchestrated by an increasing level of estrogen. This phase is interrupted by ovulation, a phenomenon associated with a switch in sex hormones production from estrogen dominant to progesterone, which is responsible for differentiation of endometrial cells, and in general for preparation for embryo implantation and potential pregnancy. After ovulation, the endometrium enters the secretory phase. Following, the absence of fertilized ovum and gradual drop off in a level of steroid hormones promote endometrial instability, thereby in effect triggering the menstruation [186,187].

### 5.2. TGFβ Isoforms Expression Pattern in Human Endometrium

In human endometrium, the expression of all three TGFβ isoforms has been observed by many research groups [188,189,190,191,192,193]. Although according to that, TGFβs expression in human endometrium is conflicting. An unquestionable remark is that TGFβ1 and TGFβ3 are both reported in glandular and stromal cells [188,189,190,194], whereas the TGFβ2 expression is more evident in the stromal compartment [188,189,194,195]. Considering the menstrual cycle phases, the TGFβ ligands are commonly expressed. However, TGFβ1 and TGFβ2 expression is not affected by the cycle phase, contrary to TGFβ3 isoform, which demonstrates maximal glandular production during the late secretory phase [191,196].

Furthermore, TGFβ type I and type II receptors are localized in both endometrial compartments, i.e., stromal and epithelial. Interestingly, TGFβRII shows a much higher expression level as compared to TGFβRI [194,197]. This observation might support the suggestion that TGFβRI is a significant player responsible for the limitation of TGFβ signal transduction in the endometrium. The TGFβ signaling could not be possible without the engagement of TGFβ receptors type III in this pathway. As it has been shown, betaglycan expression is observed in epithelial, as well as in stromal cells, where its immunostaining is predominant [198]. However, in epithelial glandular cells, betaglycan staining is abundant in the basal and apical borders of the cell, while the central part presents reduction or no staining. Worth noting is that betaglycan expression at the transcriptomic level tends to be elevated during the early secretory phase, which does not affect its protein level. An increased betaglycan gene expression in the mid-secretory phase, together with an elevated endometrial level of α-inhibin, correlates with a lower chance of achieving pregnancy with in vitro fertilization [199]. Additionally, strong immunoreactivity of betaglycan appears to consistently and diffusely locate on vascular endothelial cells lining small arteries, capillaries, and veins, both in the endometrium and in the myometrium [198,200].

Interestingly, the expression of another important TGFβ co-receptor, an endoglin in human endometrium, is associated with the vascular endothelium, where it is regarded as a specific marker of activated endothelial cells. Its angiogenic potential is pronounced as endoglin positive staining is observed in eutopic and ectopic endometrium of women with diagnosed endometriosis, with even higher intensity in the eutopic than ectopic endometrium. Conversely, endoglin immunoreactivity is not observed in the endothelial cells of microvessels of a normal endometrium [201]. On the other hand, one research group demonstrated endoglin reactivity in arterioles in comparison to veins, and endoglin moderate and intensive staining during early proliferative and early secretory phases of the menstrual cycle [202].

In summary, the TGFβ isoforms are abundantly expressed in endometrial compartments together with their dedicated receptors and co-receptors. As shown, their expression is varied and depends on the layer of the endometrium (functional vs. basal). Moreover, cyclic changes in the distribution of TGFβ signaling components are observed throughout the menstrual cycle. These observations implicate a potential role of the TGFβ pathway in maintaining endometrial homeostasis. The errors in TGFβ pathway counterparts result in the development and progression of different human pathologies related to the uterus. Impaired TGFβ signaling is noted in many disorders, such as infertility, recurrent miscarriages, uterine-placental dysfunction, endometriosis, and endometrial cancer [203,204,205,206]. The particular role of TGFβ isoforms in the etiology of endometrial cancer is provided in the following paragraphs.

## 6. Involvement of TGFβ Signaling in Endometrial Cancer Development and Progression—What We Know from Clinical Studies

### 6.1. From Tumor Suppressor to Tumor Promoter and Metastasis

In addition to the identification of well-defined risk factors of endometrial cancer development, and described in the Introduction paragraph, the mechanism of endometrial carcinogenesis remains poorly understood [10,11,12,13,14,15,16]. Many researchers link endometrial tumorigenesis with deregulations and/or disruptions in the TGFβ pathway. The TGFβ ligands play a multifaceted role in cells, tissues, and organisms physiology. Similar to a conductor, TGFβs orchestrate a whole variety of cellular processes, including proliferation, differentiation, migration, and apoptosis, in general, molecular events engaged in embryonal development, tissue homeostasis and regeneration, angiogenesis, immunomodulation, ECM remodeling, or motility [24,207]. The alteration in at least one of the above-mentioned processes may lead to cancer development and progression. The most important fact concerning TGFβ signaling during carcinogenesis, and which should be emphasized, is that TGFβ ligands play a dual role in neoplastic transformation, both as a tumor suppressor and tumor-promoting factor. Furthermore, different cancer studies revealed that TGFβs act as an anticancer agent at the early stages of carcinogenesis, whilst in the late stages, they promote cancer cell survival, invasion, and metastasis [21,208].

### 6.2. TGFβ Isoforms’ Deregulation in Endometrial Cancer

To elucidate the involvement of TGFβ pathway alterations in endometrial carcinogenesis, several research groups evaluated TGFβ isoforms expression during neoplastic transformation of human endometrium by comparison of how it has been disrupted in the following stages of cancer progression, ranging from normal endometrium, through simple and complex hyperplasia, to endometrial carcinomas [189,209,210,211,212,213] (Table 1). It has been observed that increased TGFβ1 expression is an early event in neoplastic transformation of the endometrium, as it is reported in simple hyperplastic endometrium when compared with normal tissue. Furthermore, it undergoes stepwise upregulation during the progression from simple to complex hyperplasia, but with no further increase in immunoreactivity in the case of endometrial cancer [189] (Table 1). However, the observed variation in TGFβ1 protein expression is not accompanied by elevated mRNA level, which is dramatically reduced in endometrial carcinomas [213,214]. When compared, endometrial cancer displays higher mRNA expression of TGFβ1 than adjacent non-cancerous endometrium [215]. On the other hand, the aberrant TGFβ1 mRNA level correlates with prolonged disease-free survival with no regard to the tumor stage, grade, size, subtype (endometrioid-type vs. clear-cell carcinoma), myometrial invasion, lymphovascular invasion, and recurrence [216]. However, based on the combined mRNA expression levels of TGFβ1, FXYD5/dysadherin, PAI-1, tumor necrosis factor-α (TNF-α), and patients’ survival dataset from The Cancer Genome Atlas-Uterine Corpus Endometrioid Cancer study (TCGA-UCEC), there two groups of patients can be distinguished, described respectively as low- and high-risk of poor survival outcome, where the latter is characterized by the highest mRNA levels of the above-mentioned genes [217] (Table 1). Observed positive correlation between mRNA levels of TGFβ1 and TGFβ1-induced expression of FXYD5/dysadherin is associated with pronounced invasive phenotype of endometrial cancer, depicted by myometrial invasion > 50%, grade 3, and intermediate/high risk of recurrence [217]. Similarly, in the case of uterine carcinosarcoma, relative TGFβ1 transcript level has shown a trend towards higher expression in patients with tumor recurrence [218]. The malignant endometrial cells also present an abolished ability to activate latent TGFβs in what was confirmed in endometrial cancer explants [209]. As demonstrated, the TGFβ1 isoform displays a paracrine mode of action, thus regulating endometrial cell proliferation. The TGFβ1 seems to mediate communication between endometrial carcinoma and stromal cells, and its deregulated expression may confer with endometrial carcinogenesis [209,210] (Table 1).

According to the literature data, little is known about the expression levels of TGFβ2 and TGFβ3 isoforms during endometrial carcinogenesis. As reported, TGFβ2 and TGFβ3 present increased immunoreactivity in endometrial carcinoma as compared to the normal endometrium, and their expression reflects the levels observed in the case of complex endometrial hyperplasia in both the glandular and stromal constituents of the endometrium [189]. Comparable TGFβ2 and TGFβ3 mRNA expression with that noted in the case of TGFβ1 encoding gene is observed, and is characterized by predominant mRNA expression in the stromal compartment [189]. Unfortunately, there is a lack of any data concerning TGFβ2 and TGFβ3 deregulation in the context of clinicopathological parameters of endometrial cancer.

### 6.3. TGFβ Canonical Receptors Loss in Endometrial Cancer

The proper TGFβ signaling could not occur without ligand-specific TGFβRs, of which disrupted expression or functioning have been reported in endometrial cancer. The ALK5 (TGFβR1) and TGFβR2 at the message level are profoundly decreased in cancerous tissue in comparison with normal endometrium. Although, at first glance, the published data could lead to misinterpretation. The TGFBR1 and TGFBR2 genes expression levels are strongly dependent on the use in the comparison control group (secretory, proliferative, post-menopausal endometrium or adjacent non-cancerous endometrium vs. endometrial cancer samples obtained from patients’ tissue-matched or patients’ tissue-unmatched studies, as well as different inclusion criteria of patients to the control group) and/or applied experimental methodology [209,211,219,220,221] (Table 1). The analysis of TGFβR1, both at mRNA and protein levels, has shown a significant decrease in endometrial carcinoma when compared with proliferative endometrium and assessed by in situ hybridization (ISH) and immunohistochemical staining (IHC), or reverse-transcription-PCR (RT-PCR) performed with mRNAs derived from primary cell cultures [209]. On the other hand, an increased TGFβR1 transcript level is observed in patients diagnosed with endometrial adenocarcinoma at their post-menopausal age (60–72 years) in comparison to proliferative and secretory endometrium obtained from young women (35–41 years) [219]. Altered TGFβR1 mRNA expression seems to be unassociated with the clinicopathological features. i.e., tumor grade, FIGO classification, and depth of myometrial invasion of studied cancer cases [211,220] (Table 1).

Some scientific reports have documented the involvement of TGFβR2 in endometrial carcinogenesis, which similarly to TGFβR1 presents a lower expression level than observed in normal proliferative endometrium [209,221]. Furthermore, the loss of its expression at the messenger level is followed by a decrease of TGFβR2 protein [209,222,223]. However, some research groups report contradictory results indicating the lack of a straightforward relationship between transcriptomic and proteomic levels of TGFβR2, where an elevated protein expression contrasts with mRNA down-regulation [213,220]. Interestingly, the divergent expression profile of the TGFβR2 transcript in endometrial cancer has been observed in relation to the patient’s age at diagnosis. As reported, both normal and tumorous endometria obtained from women in their postmenopausal age are characterized by significantly higher mRNA levels as compared with respective premenopausal-related specimens [221]. It is worth pointing out that TGFβR2 protein expression associates with the pronounced malignant phenotype of endometrial cancer, assessed by myometrial invasion. Unlike cancers infiltrating below the half of myometrial wall thickness, more advanced cancers presenting myometrial infiltration to a greater extent than the half of myometrium are characterized by a significant increase of TGFβR2 protein expression [211] (Table 1).

Impaired TGFβRs expression in endometrial cancer can be a result of their transcriptional down-regulation, an increased receptor degradation, and/or deregulated trafficking to the cell membrane. An additional mechanism responsible for observed alterations in TGFβRs expression can be addressed to mutational inactivation, occurring in their genes. When comparing to the other tumor types, mutations leading to the TGFβRs loss or inactivation in endometrial cancer are relatively infrequent [206]. There is only one scientific report documenting TGFBR1 gene mutations in sporadic endometrial tumors, however in a low extent equal to 2.6% (1/39) of analyzed cases and compared with patient-matched endometrial tissue from histologically confirmed tumor-free areas. Observed mutations have included a 3-bp deletion replacing Arg and Glu at codon 237 and 238 by Lys in exon 4; and an in-frame 9-bp deletion in the (GCG)_9_ microsatellite region in exon 1, resulting in loss of three alanine residues corresponding to the boundary between the signal sequence and the mature extracellular domain of the protein [224] (Table 1). Genetic alterations are better defined in the case of the TGFBR2 gene. Studies concerning sporadic endometrial carcinoma revealed a silent polymorphism (AAC→AAT) at codon 389 in the TGFBR2 gene in 44% of analyzed cancer cases [224]. Furthermore, the sequence-changing single mutations were observed in 17% of studied carcinomas and were predominantly located within the kinase domain of TGFβR2. The limited number of cancer cases have displayed two missense mutations in extracellular and C-terminal regions of functional TGFβR2 protein [224]. The loss of TGFβR2 expression both at mRNA and protein level can be attributed to the inactivating somatic mutation in specific 5’ poly(A) regions of the mRNA (located in the extracellular domain) and termed in the literature as the big A tract mutation in TGFβ receptor type II (BAT-RII) [209] (Table 1). The above-mentioned genetic alteration includes frameshift mutation in mononucleotide stretch of 10 consequent adenines (A10) repeats. The truncated TGFβR2 proteins of 161, 129, or 130 amino acids are produced, respectively when adenine is inserted or deleted creating −1, −2, or +1 frameshift mutations in comparison to the wild type TGFBR2 sequence [225]. As it is observed in different studies, BAT-RII mutations occur with variable frequency ranging from 24% to 50% [209,226,227]. These frameshift mutations are frequently associated with microsatellite instability (MSI), because the short poly(A) tract in the TGFBR2 coding sequence makes it prone to mutations [228,229]. The increased number of mutations observed in MSI-related endometrial cancer is a result of MLH1 gene promoter hypermethylation, which leads to a deficit of DNA mismatch repair system (dMMR), and in effect the accumulation of genetic errors [228,230] (Table 1). Interestingly, endometrial cancers considered as MSI-high, i.e., presenting alteration in ≥40% of analyzed microsatellite markers, are relatively rare events (5%), particularly when compared with other MSI-prone types of cancers originated from the colon (58%) and stomach (80%) [229]. This seems to stay in line with an analysis of MSI-related mutations in the TGFBR2 gene in patients with diagnosed HNPCC, a disease in which the colorectum and uterine endometrium are the two most commonly affected organs. The HNPCC patients display TGFBR2 mutations more frequently in colorectal than endometrial cancers (88% vs. 25%), and the main difference between these tumor types is PTEN instability, which seems to be characteristic for uterine tumorigenesis in this pathology [231] (Table 1). Given together, distinct instability profiles in HNPCC-related colorectal and endometrial cancers indicate organ-specificity despite similar molecular predisposition.

### 6.4. Deregulation of TGFβ Signaling at the Level of Smad Proteins

TGFβ pathway governs cell and tissue homeostasis at multiple levels via the regulation of opposing molecular processes. To orchestrate that, TGFβ signaling demands the activity and precise localization (cytosolic vs. nuclear) of intracellular downstream Smad proteins. In contrast to the other cancer types, the knowledge concerning the alterations of Smad expression in endometrial cancer is limited [232]. Based on the literature findings, Smad proteins serve as a tumor suppressor, and their inactivation or deregulation may contribute to the development and progression of uterine neoplasms. The literature data indicate that SMAD2 and SMAD4 expression is not altered in endometrial cancer when compared to normal endometrium [209,233] (Table 1). In addition to that observation, significantly down-regulated transcript levels of these genes are noted in the case of infiltrating endometrial tumors (less and more than half of the myometrial wall thickness). Although, no such relation is observed for the SMAD3 gene [211]. On the other hand, a study has been published presenting that decreased mRNA levels of SMAD2, SMAD3, and SMAD4 are frequent events, respectively in 71.4%, 78.6%, and 78.6% of analyzed endometrial cancer samples. Observed down-regulation is correlated with clinical data and patient outcome. SMAD2 and SMAD3 mRNA levels are associated with nuclear and FIGO grades, and the SMAD4 mRNA level is significantly associated with tumor size, tumor subtype, lymphovascular invasion, nuclear and FIGO grade, and disease-free survival [216] (Table 1). Observed inconsistency between these studies, may be a result of a different number of analyzed cases (39 vs. 71), as well as different histopathological subtypes included in the study cancer samples (endometrioid type vs. endometrioid, clear cell carcinoma, and serous).

Smad proteins, as cellular effectors of the TGFβ pathway, to provide downstream signalization, demand activating phosphorylation by TGFβRI receptors located in the cell membrane. Following, the activated heteromeric Smads complexes translocate to the nucleus, where together with other co-activators or co-repressors they modulate TGFβ-responsive genes. Due to that fact, the critical role in Smads protein physiology is their subcellular distribution, respectively cytoplasmic or nuclear localization. The analysis of Smads expression at the protein level in endometrial carcinoma revealed that their intracellular distribution undergoes changes during uterine neoplastic transformation. For instance, Smad4 protein expression in a cytoplasmic fraction is gradually increased in association with tumor aggressiveness and progression, evaluated by tumor grade and myometrial infiltration. In general, during cancer progression, a decreased number of samples is observed that are characterized by the presence of Smads exclusively in a nuclear fraction [211]. On the other hand, one group reported a reversed relationship between Smad4 and tumor grading, i.e., Smad4 immunohistochemical staining decreases progressively with tumor grade, however without the correlation with patient’s outcome [234]. Smad2 and Smad4 immunoreactivity in endometrial cancer is comparable with that observed in normal endometrium [209,235] (Table 1). Nonetheless, Smad2 phosphorylated from (pSmad2) staining is undetectable or weak in endometrial cancer and reduced in glandular hyperplasia compared to normal endometrium [209,236]. Unlike Smad2 and Smad4, Smad3 nuclear localization is diminished in tumor samples [233]. Conversely to the discussed Smad proteins, Smad7, which antagonizes TGFβ induced signaling, is overexpressed at mRNA level in endometrial adenocarcinoma. That upregulation strongly correlates with poor prognosis, as the median period to recurrence for the patients with high expression of Smad7 is 30 months vs. 56.3 months in the case of patients with lower levels of Smad7 [236].

Deregulation of Smads expression can be attributed to the genetic mechanisms, but they are not well studied in endometrial cancer. According to TCGA-UCEC, mutations in Smads encoding genes are relatively infrequent, unlike other cancer types with identified Smads alterations. Mutations related to Smads’ genes are observed in 11.4% of endometrioid carcinoma cases, among which 5% in SMAD2, 4.6% in SMAD3, 3.5% in SMAD4, and 6.8% in SMAD7 [7,237] (Table 1). SMAD2, SMAD4, and SMAD7 genes are located at the 18q21 locus, which is highly prone to the MSI or loss of heterozygosity (LOH) in endometrial cancer. The allelic imbalance in locus 18q21 has been identified in 16.7% (MSI) and 20% (LOH) of analyzed endometrial adenocarcinomas cases. Interestingly, reported genetic alterations seem to be characteristic for more advanced cancers, thus occurring predominantly in stages FIGO III and FIGO IV. Furthermore, LOH is exclusively identified in the SMAD2 gene, what might suggest the importance of alterations of the SMAD2 gene over the other Smads-encoding genes assigned to the 18q21 locus [238]. On the other hand, in the promoter sequence of the SMAD4 gene, one- and two-base substitutions (T→C transversion at position −154; and GG→AA transversion at position +5–6) were identified that may potentially disturb SMAD4 transcription. That region overlaps binding sites crucial for SMAD4 expression transcription factors, such as c/EBPb and GATA2. Identified substitutions probably arise as somatic mutations since they are not identified in matched normal tissue [239].

### 6.5. TGFβ Signal Modulation Can Be Altered by Impaired Co-Receptors Expression

Induced by TGFβ isoforms extracellular stimuli transduction may also be interfered at the level of signal modulation by altered expression of TGFβ co-receptors. According to the clinical studies, this step in TGFβ signaling is impaired since the expression of two TGFβ co-receptors, i.e., betaglycan and endoglin, is changed in endometrial cancer. TGFBR3 gene, which encodes betaglycan, is significantly reduced in cancer tissue, corresponding to its immunoreactivity loss. Well-differentiated tumors present weak or patchy staining with rare immunoreactivity signals localized to the epithelial glands, whereas poor-differentiated adenocarcinomas present devoid of stains in endometrial components. The lack of betaglycan in epithelial compartments of the endometrium is opposed by strong positive immunostaining of tumor vessels, which suggests that it may be a distinct player in tumor vascularization [200]. The evaluation of betaglycan expression at the transcriptomic level in the context of clinicopathological features of studied material indicates that its loss occurs as an early event in neoplastic transformation of human endometrium [222] (Table 1). Further studies have revealed that observed betaglycan down-regulation results from different genetic mechanisms, including LOH and single nucleotide polymorphisms (SNPs) in the locus of the TGFBR3 gene [240,241]. LOH, assessed using three microsatellite markers (D1S188, D1S435 and D1S1588), is a relatively frequent event and occurs in 52% of all analyzed primary endometrial carcinomas (Table 1). An additional mechanism with potential impact on the declined expression of the TGFBR3 gene involves three intronic SNPs, i.e., rs12566180 (c.-114 + 2392C > T) and rs2296621 (c.2285 − 99G > T), which correlate with TGFBR3 transcript loss in endometrial cancer. Moreover, these SNPs and an additional one, rs6680463 (c.-114 + 7008C > G), are significantly associated with increased risk of endometrial cancer, respectively in the case of genotypes CT (rs12566180; OR = 2.22; 95% CI = 1.15–4.30; *p* = 0.0177), GC (rs6680463; OR = 2.34; 95% CI = 1.20–4.53; *p* = 0.0120) and TT (rs2296621; OR = 6.40; 95% CI = 1.18–34.84; *p* = 0.0317) [241] (Table 1). In the context of clinicopathological parameters, only rs2296621 seems to favor an increased tumor aggressiveness evaluated by the WHO grading system (G3 vs. G1/2, GT—OR= 4.04; 95% CI = 1.56–10.51; *p* = 0.0026; T—OR= 2.38; 95% CI = 1.16–4.85; *p* = 0.0151).

The last discussed component of the TGFβ pathway, which significantly influences signal transduction and promotes carcinogenesis of the endometrium, is endoglin. Endoglin is the most studied TGFβ co-receptor in endometrial carcinogenesis. Based on the literature data, its changed expression can be regarded as a valuable diagnostic and prognostic marker of tumor behavior. In endometrial cancer, endoglin displays significant protein up-regulation, with concomitant not altered mRNA expression [223,242] (Table 1). Due to the confirmed angiogenic potential of endoglin, its staining is preferentially observed in proliferated endothelial cells. Endoglin’s assessment, together with microvessel density (MVD), suggests that it can be regarded as a promising diagnostic marker in women with endometrial cancer. Furthermore, its prognostic value seems to be complementary or even better to the routinely applied molecular markers, such as CD34, used to visualize tumor advancement [242,243,244,245,246,247,248] (Table 1). Interestingly, there are no significant differences in endoglin staining between endometrial polyps and endometrial adenocarcinomas, which supports the fact that neovascularization is associated both with hyperplastic and neoplastic lesions of the endometrium [249]. On the other hand, the preoperative serum levels of endoglin in patients with endometrial carcinoma show poor performance as a diagnostic marker of tumor metastasis [250].

## 7. TGFβ-Based In Vivo and In Vitro Studies on Endometrial Carcinogenesis

### 7.1. TGFβ-Mediated Tumor-Suppressive Program in Endometrial Carcinogenesis

On the contrary to other cancer types, until recently, there was a lack of an elegant mouse model of endometrial carcinogenesis involving TGFβ signaling. According to recent findings by the Matzuk group, in which the Cre-loxP approach was applied for the generation of mice with conditional knock-out of ALK5 or Smad2/3 in the uterus, the TGFβ pathway appeared to be a key player not only in the uterine physiology but also as an essential factor contributing to the cancer development of this organ. In the studies mentioned above, genes encoding ALK5 or Smad2/3 were deleted both in the uterine epithelium, stroma, and myometrium, using progesterone receptor Cre recombinase [251,252]. As observed, mice with abrogated ALK5 develop enhanced endometrial oncogenesis only in female mice being continuously mated with fertile males, whereas nulliparous females with Alk5 conditional knock-out stay cancer-free. This suggests that ALK5 is crucial for uterine function as a necessary factor for postpartum endometrial repair mediated by TGFβ signaling [252]. The ablation of ALK5 in mice leads not only to malignant transformation of the endometrium but also triggers its increased metastatic potential. However, an elevated aggressiveness of endometrial cancer under the conditional knock-out of Alk5 is observed only in the case of concomitant PTEN abrogation. The observed preferential metastatic site is the lung, which results in a dramatically reduced lifespan. The accelerated tumor progression is a result of elevated secretion of proinflammatory chemokines, induction of cancer cell motility manifested by myometrial invasion and disruption, as well as impaired tumor microenvironment via recruitment of tumor-associated macrophages [253]. Given together, it indicates the prominent role of the TGFβ-PTEN axis in the suppression of endometrial cancer progression. The exclusive uterine epithelial loss of PTEN with intact stromal expression is insufficient to induce endometrial carcinogenesis in a mouse model since it only intensifies cell apoptosis through elevated levels of TGFβ and activation of downstream effectors Smad2/3 in the uterine stroma [254].

Another vital element of the TGFβ pathway, whose deregulation participates in maintaining uterine homeostasis, is the Smad2/3 complex. Its engagement in endometrial cancer development and progression has been uncovered by Kriseman et al., who generated and described Smad2 and Smad3 double-conditional knocked-out mice [251]. Using that approach, they provided evidence supporting the role of Smad-dependent TGFβ signaling in endometrial tumorigenesis. Mice with knocked-out Smad2/3 complex are infertile due to the hyperplastic phenotype manifested at the pubertal-onset. Observed endometrial hyperproliferation undergoes gradual progress and ultimately develops into massive endometrioid-type uterine cancer with 100% lethality up to 8 months postnatal. Interestingly, the dramatic mortality can be effectively reduced by both-sided ovariectomy by six weeks of age. This fact strongly emphasizes the hormone-dependent character of Smad2/3-mediated signaling in uterine physiology [251]. As reported in this study, uterine neoplastic transformation is associated with the downregulation of genes involved in steroid biosynthesis, increased expression of inflammatory response genes, and altered cell cycle checkpoint genes expression. In addition to these genes, RNA-sequencing indicated that the ablation of Smad2/3 affects the crosstalk between TGFβ and BMP signaling, as it activates BMP target genes involved in cell growth and angiogenesis [251]. Intriguingly, TGFβ and BMP interplay remains unclear. The potential explanation includes direct suppression of the BMP pathway by activated Smad2/3, or BMP up-regulation may occur as a compensational mechanism in response to the loss of Smad2/3. Furthermore, BMP signaling shares type II receptors, i.e., ActRIIa, ActRIIB, with activins [255].

The results obtained in discussed mouse models support that the loss of growth inhibitory function of TGFβ signaling contribute to endometrial carcinogenesis presented by Parekh et al. In that study, the primary cultures of endometrial epithelial cells derived from normal proliferative endometrium underwent dose-dependent and maximal growth inhibition up to 55% ± 5.3% when treated with 10 pM TGFβ1, whereas endometrial epithelial cells derived from endometrial carcinomas stayed unresponsive to TGFβ1 isoform [209]. Together with further studies, unresponsiveness to TGFβ1 isoform in the case of endometrial cancer suggests that escape from the inhibiting impact of TGFβ signaling occurs at the early stages of carcinogenesis [209]. On the other hand, the microarray gene-expression profiling of the high-risk recurrence endometrial carcinomas undoubtedly revealed a prominent role of TGFβ1 signaling in acquiring an aggressive phenotype. The TGFβ1 isoform initiates the invasion by activating epithelial to mesenchymal transition in HEC-1A and RL95-2 cells, a process in which epithelial cells develop invasive phenotype. The metastatic potential of tumor cells can be reversed by SB-431542, a specific TGFβ1 inhibitor, which in effect precludes further persistent endometrial carcinoma invasion [256]. Discussed studies indicate that a functional TGFβ signaling program is required for cellular homeostasis and orchestrate extracellular stimulus to protect against endometrial tumorigenesis. ALK5-Smad2/3 signaling route plays a significant suppressive branch of the TGFβ pathway in uterine pathophysiology.

### 7.2. TGFβ-Induced Tumorigenic Program in the Progression of Endometrial Cancer

On the other hand, some in vitro studies indicate indisputable involvement of TGFβ-induced signaling in the initiation of endometrial cancer invasion throughout induction of epithelial to mesenchymal transition (EMT). As confirmed using two endometrial cancer cell lines—HEC 1A and RL95 2—the acquisition of metastatic phenotype is observed upon the stimulation with TGFβ1 isoform. TGFβ1-treated cells present a decreased cell-cell contacts and the promotion of migratory structures as lamellipodia, what is accompanied by an increase of mesenchymal marker vimentin. The described impact of TGFβ1 on endometrial cells can be effectively abolished using the specific TGFβ1 inhibitor SB-431542, which restores epithelial architecture and the formation of compact colonies [256]. Recently it has been demonstrated that TGFβ1 promotes EMT via induction of phosphorylation of eukaryotic translation initiation factor 4E (eIF4E) in HEC 1A cells [257]. eIF4E is considered as an oncogene that is overexpressed in different human malignancies, and its upregulation is correlated with advanced stages of carcinogenesis [258]. Targeting eIF4E with miR-320a and miR-340-5p prevents TGFβ1-induced EMT with simultaneous down-regulation of MMP 3 and MMP-9 in HEC-1A cells [257].

Additionally, the TGFβ pathway may also enhance cell motility and invasiveness—the features of cells undergoing EMT, through another downstream target prostate apoptosis response 4 protein (Par-4). Par-4 was initially described as a tumor suppressor responsible for activating apoptosis induced by extracellular cytotoxic signals [259]. However, in the context of EMT induced by the TGFβ cascade, it paradoxically acts as a factor promoting cancer metastasis. The exogenous treatment of endometrial cells, KLE and HEC-1A, with TGFβ isoforms results in up-regulation of Par-4, both at transcriptomic and protein level [260]. What is worth underlining is that the observed increase of Par-4 occurs in a Smad-dependent, as well as a Smad-independent manner. The activation of the first route is confirmed by an elevated level of pSmad2, whereas initiation of the second one is associated with increased phosphorylation of IκB α, which is an inhibitor of NF κB signaling [260]. Among three TGFβ isoforms, TGFβ3 displays the most evident potential to induce Par-4-related EMT manifested by changed cell morphology. Furthermore, prolonged exposure to TGFβ3 isoform triggers elevated levels of EMT signature genes, such as Snail, vimentin, zinc-finger E-box binding homeobox 1, and N-Cadherin, with a concomitant decrease of Claudin-1 and E-Cadherin [260].

During endometrial cancer progression, in particular initiation of the metastatic process, a vital role is played by the tumor microenvironment serving as a reservoir of cytokines, growth factors, and other factors. Among these molecules, TGFβ1 is released directly or as cargo in exosomes released from cancer cells, and/or immunological cells trigger the transformation of normal fibroblast (NF) to cancer-associated fibroblasts (CAFs) [261]. TGFβ1-induced CAFs transition is additionally enhanced by the action of miR¬21 through the translation inhibition of Smad7 mRNA. TGFβ1-related CAFs activation can be effectively blocked by overexpression of miR¬22 or the depletion of inhibitory Smad7 [262]. CAFs, in turn, stimulate different pro-tumorigenic events, such as extracellular matrix remodeling and secretion of cytokines, further promoting cancer aggressiveness [263]. The main cytokines secreted by CAFs isolated from human endometrial cancer tissue include MCP-1, CCL5, RANTES, interleukin-6, and -8 (IL-6, IL-8) VEGF, EGF, HGF, FGF-2, as well as TGFβ1 isoform [264,265]. As demonstrated, molecules secreted by CAFs, i.e., TGFβ1, EGF, HGF, and FGF-2, potentiate the migration and invasion of endometrial cancer cells (RL-952) when administered exogenously, thus inducing lung metastasis in vivo in mouse subcutaneous xenograft assay [265]. In the case of IL-6, CAFs promote endometrial cancer cells proliferation via activation of the IL-6/STAT-3/c-Myc axis, both in vitro and in vivo models [266]. Whilst VEGF, as a powerful proangiogenic factor, is responsible for vascularization of cancer milieu promoting angiogenesis within the tumor [264].

Conditioned-culture media of CAFs significantly induces dose-dependent proliferation of primary endometrial cell cultures, as well as endometrial cancer cell lines when compared with untreated control cells or cells treated with conditioned media obtained from NF. The CAFs-associated proliferation of endometrial cancer cells occurs via the SDF-1/CXCR4 axis, which in turn activates PI3K/Akt and MAPK/ERK signaling pathways in a paracrine-dependent manner. Simultaneously, an increased secretion of MMP-2 and MMP-9 in an autocrine-dependent manner was observed [267]. CAFs contribution to the endometrial cancer cells progression can be impeded using rapamycin targeting mTOR, a downstream effector of the PI3K pathway [264].

Considering endometrial cancer risk factors, particularly prolonged exposure to estrogens that are not balanced with progesterone (that issue is discussed in the Introduction paragraph), it has been demonstrated that extended progesterone treatment effectively reduces the metastatic ability of endometrial cancer cell lines HEC-1B, RL-952 and Ishikawa [268]. This observation has been accompanied by reduced expression of vimentin and elevated level of E-Cadherin, the acknowledged markers of the EMT process. Furthermore, progesterone-treated cells present the reduction of TGFβ signaling components, i.e., Smad2/3, pSmad2/3, Samd4, ALK5, TGFβR2, and betaglycan in a time-dependent manner with a notable exception of intact ALK5 and TGFβR2 expression observed for RL-952 cells. [268]. A progesterone-related down-regulation of TGFβ pathway key players is considered as a crucial event contributing to growth inhibition and abolishing of EMT in endometrial cancer.

## 8. Conclusions and Future Perspectives

**Figure 7:**
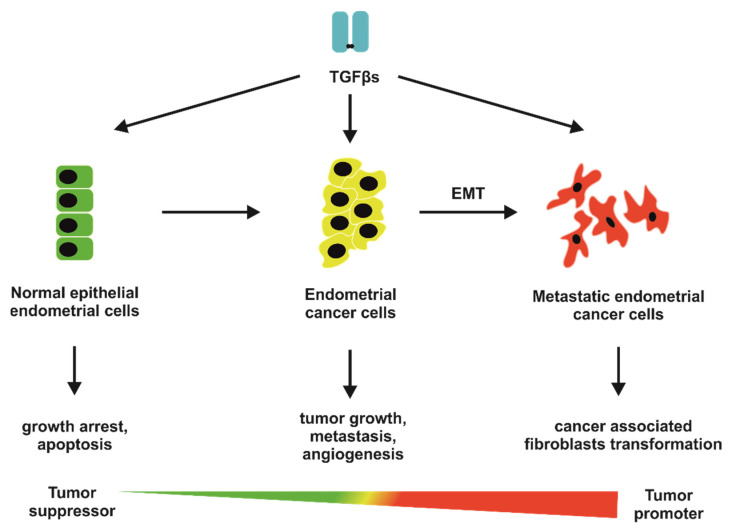
The pleiotropic role of TGFβ signaling during endometrial carcinogenesis.

The TGFβ pathway, which belongs to the signaling network in the cell, is one of the most essential cascades governing a vast of cellular processes, both in health and disease. The plethora of molecular events under control, and/or being dependent on the TGFβ signaling finds its reflection during carcinogenesis. On the contrary to the other well-known suppressors and oncogenic factors, the TGFβ pathway plays a pleiotropic role in orchestrating contradictory processes. As it is observed in the case of TGFβ-induced stimuli, TGFβ signaling contributes to the suppressor program in cancerous cells at the early stages of carcinogenesis. During the progression of neoplastic disease, the TGFβ axis supports increased cell growth and proliferation, leading to pronounced aggressiveness and invasion, thus stimulating cancer cells seeding and establishing new metastatic sites (Figure 7).

As presented in this review, TGFβ pathway components undergo deregulation in endometrial cancer. Impaired expression is observed at every level of signal transduction, beginning from signal induction by TGFβ isoforms, signal reception by plasma membrane receptors and co-receptors, and up to downstream cytosolic effector Smad proteins. All these alterations are of great potential, as diagnostic and prognostic markers of endometrial neoplastic transformation. However, further investigations need to be performed to understand the uterine cancerous transformation better and/or increase the clinical value of identified molecular mechanisms. Moreover, extensive studies would open a new perspective for establishing novel anti-cancer strategies targeting TGFβ signaling components. This issue is fundamental since to date there is a paucity of reliable molecular biomarkers dedicated to endometrial cancer diagnosis and monitoring the effectiveness of therapy. Routine treatment of this tumor type is surgery, which includes total hysterectomy with removal of fallopian tubes and ovaries. In the early stages of endometrial cancer, the majority of patients have a favorable prognosis. However, some women are at risk of disease relapse, so the crucial question is which patients would benefit from adjuvant treatment, including chemo- and/or radiotherapy [6]. An answer to this dilemma could be resolved after a better understanding of TGFβ engagement in uterine neoplastic transformation.

As discussed in this review, the engagement of the TGFβ pathway in endometrial cancer progression shows that TGFβ-induced signaling is a prominent route determining increased invasiveness and metastasis. Invasion and metastasis are considered as the main reasons for treatment failure and poor patient survival. Furthermore, the accumulation of molecular insults underlies the development of drug resistance to hitherto successfully administered drugs. In effect, there is a need for modification or developing new therapeutic strategies, which could provide a better, and personalized approach in treatment adjusted not only to the cancer type but to the particular patient. 

The final issue is the identification of molecular events responsible for the switch of TGFβ-induced signaling from tumor suppressor to tumor promoter. Unfortunately, so far there is a lack of such evidence during endometrial carcinogenesis. Taken together, the TGFβ pathway is an underestimated signaling cascade in human endometrium, thereby there is a need for more extensive research covering the involvement of TGFβ in uterine neoplastic transformation. To confirm that fact, there has been only one drug ongoing clinical trial of TGFβ antagonist in endometrial cancer, i.e., tasisulam. Tasisulam is a TGF-β/TGF-β type I receptor kinase (ALK5) inhibitor assigned to clinical trials in patients with refractory or malignant solid tumors. It has been tested in two phase I clinical trials. The first one included only one patient with endometrial cancer, whereas the second phase Ib clinical trial involved 13 patients with ovarian, uterine, endometrial, and cervical cancers. However, the exact number of endometrial cancer cases has not been indicated in the latter trial [269].

## Figures and Tables

**Table 1:** Altered canonical TGFB signaling components in endometrial cancer.

TGFβ Pathway Component	Tissue or Cells Type	Changes	Comments	Ref.
**TGF** **β isoforms**				
**TGF** **β1**	Simple/complex hyperplasia, endometrial cancer	Protein increased		[189]
	Endometrial cancer	mRNA decreased	Cancer vs. normal	[213,214]
	Endometrial cancer	mRNA increased	Cancer vs. adjacent non-cancerous tissue	[215]
	Endometrial cancer	mRNA decreased	Correlation with disease-free survival	[216]
	Endometrial cancer	mRNA increased	Correlation with high-risk of poor survival outcome (invasive phenotype) when combined with other markers	[217]
	Uterine carcinosarcoma	mRNA increased	In patients with tumor recurrence	[218]
	Endometrial cancer (primary cell cultures)	Loss of latent TGFβ activation	Cancer primary cell culture vs. primary cell culture of proliferative endometrium	[209]
**TGF** **β2**	Simple/complex hyperplasia, endometrial cancer	Protein increased		[189]
**TGF** **β3**	Simple/complex hyperplasia, endometrial cancer	Protein increased		[189]
**TGF** **β receptors**				
**TGF** **βR1** **(ALK5)**	Endometrial cancer	mRNA and protein decreased	Cancer vs. normal proliferative endometrium	[209]
	Endometrial cancer	mRNA increased	Cancer obtained from postmenopausal women (60–72 yo) vs. proliferative and secretory endometrium from young women (35–41 yo)	[219]
	Endometrial cancer	Mutation	2.6% of analyzed cancer cases	[224]
**TGF** **βR2**	Endometrial cancer	mRNA and protein decreased	Cancer vs. normal proliferative endometrium	[209,221,222,223]
	Endometrial cancer	mRNA decreased, protein increased		[213,220]
	Endometrial cancer	mRNA increased	Correlation with patients’ age at diagnosis (postmenopausal vs. premenopausal)	[221]
	Endometrial cancer	Polymorphism/mutations	44% (AAC→AAT at codon 389) and 17% (single mutations within kinase domain) of analyzed cancer cases	[224]
	Endometrial cancer	Mutation	BAT-RII frameshift mutation ranging from 24% to 50% of analyzed cancer cases	[209,226,227]
	Endometrial cancer	MSI	Associated with dMMR occurring in 5% of analyzed cancer cases	[228,229,230]
	Endometrial cancer in HNPCC patients	MSI/mutations	25% of analyzed cancer cases	[231]
**Smads**				
**Smad2**	Endometrial cancer	mRNA decreased	Correlated with myometrial infiltration (<1/2 vs >1/2 of myometrial wall thickness	[233]
	Endometrial cancer	mRNA decreased	Cancer vs. normal (71.4% of analyzed cancer cases), correlation with nuclear and FIGO grade	[216]
	Endometrial cancer (TCGA-UCEC)	Mutations	5% of analyzed cancer cases	[7,237]
	Endometrial cancer	LOH/MSI	20% (LOH) and 16.7% (MSI) of analyzed cancer cases	[238]
**Smad3**	Endometrial cancer	mRNA decreased	Cancer vs. normal (78.6% of analyzed cancer cases), correlation with nuclear and FIGO grade	[216]
	Endometrial cancer (TCGA-UCEC)	Mutations	4.6% of analyzed cancer cases	[7,237]
**Smad4**	Endometrial cancer	mRNA decreased	Correlated with myometrial infiltration (<1/2 vs. >1/2 of myometrial wall thickness	[233]
	Endometrial cancer	mRNA decreased	Cancer vs. normal (78.6% of analyzed cancer cases), correlation with tumor size, subtype, lymphovascular invasion, nuclear and FIGO grades, and disease-free survival	[216]
	Endometrial cancer (TCGA-UCEC)	Mutations	3.5% of analyzed cancer cases	[7,237]
	Endometrial cancer	Mutations	T→C transversion at position −154; and GG→AA transversion at position +5–6	[239]
**Smad7**	Endometrial cancer	mRNA increased	Cancer vs. normal, correlation with poor prognosis (median period to recurrence in patients with high expression 30 months vs. 56.3 months in patients with lower expression)	[236]
	Endometrial cancer (TCGA-UCEC)	Mutations	3.5% of analyzed cancer cases	[7,237]
**TGF** **β co-receptors**				
**Betaglycan (TGF** **βR3)**	Endometrial cancer	mRNA and protein decreased	Cancer vs. normal	[200]
	Endometrial cancer	mRNA decreased	Correlation with clinicopathological features of studied cancer cases	[222]
	Endometrial cancer	LOH	52% of analyzed cancer cases (microsatellite markers D1S188, D1S435, and D1S1588)	[240]
	Endometrial cancer	SNP	Correlation with decreased mRNA expression of betaglycan	[241]
**Endoglin** **(CD105)**	Endometrial cancer	Protein increased	Cancer vs. normal, correlation with tumor advancement related to angiogenesis	[223,242,243,244,245,246,247,248]

## Data Availability

Not applicable.
